# iPSC-based modeling of preeclampsia identifies epigenetic defects in extravillous trophoblast differentiation

**DOI:** 10.1016/j.isci.2024.109569

**Published:** 2024-03-25

**Authors:** Robert Morey, Tony Bui, Virginia Chu Cheung, Chen Dong, Joseph E. Zemke, Daniela Requena, Harneet Arora, Madeline G. Jackson, Donald Pizzo, Thorold W. Theunissen, Mariko Horii

**Affiliations:** 1 Department of Pathology, University of California San Diego, La Jolla, CA 92093, USA; 2 Sanford Consortium for Regenerative Medicine, University of California San Diego, La Jolla, CA 92093, USA; 3 Center for Perinatal Discovery, University of California San Diego, La Jolla, CA 92093, USA; 4 Department of Developmental Biology and Center of Regenerative Medicine, Washington University School of Medicine, St. Louis, MO 63110, USA

**Keywords:** Molecular biology, Cell biology, Omics, Transcriptomics

## Abstract

Preeclampsia (PE) is a hypertensive pregnancy disorder with increased risk of maternal and fetal morbidity and mortality. Abnormal extravillous trophoblast (EVT) development and function is considered to be the underlying cause of PE, but has not been previously modeled *in vitro*. We previously derived induced pluripotent stem cells (iPSCs) from placentas of PE patients and characterized abnormalities in formation of syncytiotrophoblast and responses to changes in oxygen tension. In this study, we converted these primed iPSC to naïve iPSC, and then derived trophoblast stem cells (TSCs) and EVT to evaluate molecular mechanisms underlying PE. We found that primed (but not naïve) iPSC-derived PE-EVT have reduced surface HLA-G, blunted invasive capacity, and altered EVT-specific gene expression. These abnormalities correlated with promoter hypermethylation of genes associated with the epithelial-mesenchymal transition pathway, specifically in primed-iPSC derived PE-EVT. Our findings indicate that abnormal epigenetic regulation might play a role in PE pathogenesis.

## Highlights

• Primed PE-iPSC-derived EVT showed abnormal EVT marker expression and invasion
• EMT genes showed low expression and promotor hypermethylation in primed PE-EVT
• PE-EVT phenotype was lost when converted to naïve iPSC, then differentiated to EVT
• Established an *in vitro* PE model that recapitulates abnormal EVT characteristics

## Graphical abstract

**Figure:**
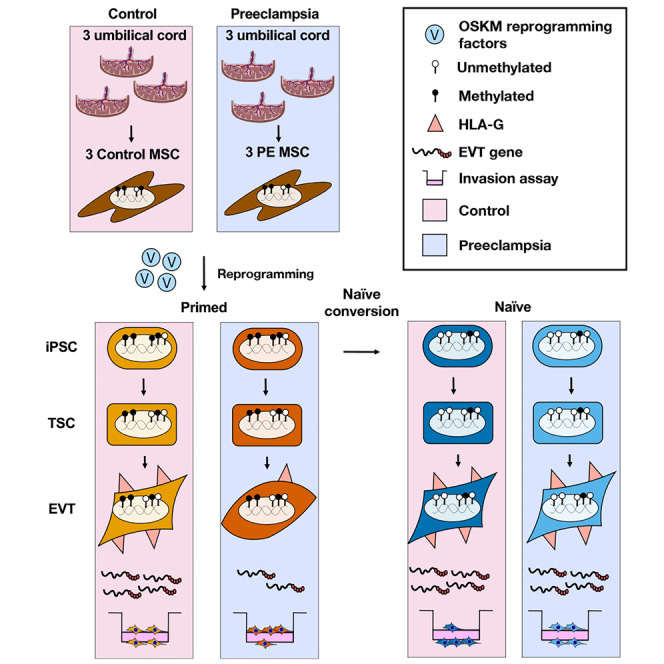

## Introduction

Preeclampsia (PE) is a new-onset hypertension with proteinuria and/or evidence of significant end organ dysfunction, appearing after 20 weeks of gestation.^1^^,^^2^ PE affects up to 8% of pregnancies, and confers an increased risk of morbidity and mortality, with increased risk of long-term consequences for developing metabolic and cardiovascular disease to both mother and baby.^3^^,^^4^ Currently, the only treatment for this disease is delivery of the placenta, leading to medically necessitated preterm birth.^2^^,^^5^ The underlying pathophysiology of PE is incompletely understood, but the “two-stage theory”, currently the most accepted hypothesis of the root cause of this disease, points to abnormal trophoblast differentiation as the underlying cause.^5^

Trophoblast, the epithelial cell of the placenta, consists of three main cell types. Cytotrophoblasts (CTBs), located in the inner layer of the villi, are self-renewing trophoblast progenitor cells, which terminally differentiate into two types of trophoblast: syncytiotrophoblasts (STB), which mediate nutrient/gas exchange, synthesize key pregnancy hormones, and protect against pathogens; and extravillous trophoblasts (EVTs), which anchor the placenta to the uterine wall through invasion, and remodel spiral arterioles to establish blood supply from the mother to the fetus.^6^^,^^7^ The “two-stage theory” states that PE is initiated by abnormal EVT differentiation leading to insufficient spiral artery remodeling/poor placentation and development of ischemia-reperfusion injury (first stage), subsequently leading to secretion of antiangiogenic factors and stimulation of vascular inflammation, concluding with maternal systemic disease (second stage).^5^ Abnormal EVT differentiation and poor vascular remodeling correspond to histopathological findings in the placenta, described as decidual arteriopathy, characterized by retention of the vascular muscle wall, perivascular chronic inflammation, fibrinoid necrosis, and/or foamy macrophages in the vessel wall (atherosis).^8^^,^^9^^,^^10^^,^^11^^,^^12^^,^^13^ These lesions interrupt blood flow into the intervillous space and lead to ischemia/reperfusion injury of chorionic villi, which then manifests histologically as accelerated villous maturation (e.g., increased syncytial knots), distal villous hyperplasia, and villous infarction.^14^ Collectively, these histological findings are called maternal vascular malperfusion (MVM), one of the four major patterns of placental injury recently described by Redline et al. (2021).^9^ MVM appears to be dominant in early-onset PE with fetal growth restriction,^9^^,^^10^^,^^15^^,^^16^^,^^17^^,^^18^ which is clinically the most severe type of PE and is commonly associated with the worst adverse maternal and neonatal outcomes.^19^^,^^20^

The difficulty in studying the detailed cellular and molecular mechanisms of this disease is in part due to the lack of an appropriate model system. Over the past two decades, multiple groups have shown that human pluripotent stem cells (hPSCs), both embryonic (ESC) and induced pluripotent stem cells (iPSCs), can be differentiated into trophoblast, and are able to be used as a model to study both normal and disease trophoblast.^21^^,^^22^^,^^23^^,^^24^^,^^25^^,^^26^^,^^27^^,^^28^^,^^29^^,^^30^^,^^31^^,^^32^^,^^33^^,^^34^^,^^35^^,^^36^^,^^37^ Our group and others have previously reported a PE-iPSC derived trophoblast model using a BMP4-based trophoblast differentiation protocol, and found that PE-iPSC-derived trophoblast show abnormalities in oxygen response mechanisms.^31^^,^^32^ We also found that these phenotypic changes were associated with DNA methylation changes, suggesting microenvironmental factors may be involved in these responses,^32^ and lead to epigenetic modifications in the placenta.^38^^,^^39^^,^^40^ Despite these results, the protocols used for trophoblast differentiation of PE placenta-derived iPSC in these studies^31^^,^^32^ were suboptimal to characterize EVT differentiation.^41^ However, recently established culture conditions for the derivation of *bona fide* human trophoblast stem cells (TSCs) have enabled the maintenance of TSC lines and greatly improved EVT differentiation capabilities.^42^

TSC are bipotent, self-renewing cells originally derived from blastocysts and early gestation placenta.^42^ The media developed for primary TSC derivation and culture have been applied to hPSC to create hPSC-derived TSC, including by our group.^35^^,^^36^^,^^37^^,^^41^^,^^43^^,^^44^^,^^45^^,^^46^^,^^47^ Early reports claimed that trophoblast stem cell derivation is limited to hPSC in the naïve- (pre-implantation epiblast-like) pluripotent state,^43^^,^^44^^,^^45^^,^^46^^,^^47^ but more recently, others (including our group) have shown that treatment of primed- (post-implantation epiblast-like) hPSC with bone morphogenetic protein 4 (BMP4), followed by culture in TSC media, allows for conversion to *bona fide* TSC.^35^^,^^36^^,^^37^^,^^41^^,^^48^ These recent hPSC-derived TSC protocols have overcome previous limitations,^30^^,^^32^ such as the inability to maintain TSC populations in culture, and a low efficiency of EVT differentiation. TSC derived from both naïve and primed hPSC demonstrate similar characteristics, including expression of CTB markers, the capacity for terminal trophoblast differentiation, *ELF5* promoter hypomethylation, and loss of classical HLA molecules (HLA-A and -B).^35^^,^^36^^,^^37^^,^^43^^,^^44^^,^^45^^,^^46^^,^^48^ However, unlike “primed” TSC, “naïve” TSC also show high expression of chromosome 19 miRNA cluster (C19MC), even higher than that of primary TSC,^46^ whereas expression levels in “primed” TSC are lower than that of primary hTSC.^35^^,^^45^^,^^46^ This is not surprising, as the C19MC is known to be an imprinted locus, and naïve conversion of hPSC results in loss of over 70% of genomic DNA methylation, including a loss of methylation at imprinted regions of the genome.^49^^,^^50^ While conversion of naïve hPSC to TSC appears to involve restoration of some trophoblast-specific imprinting,^44^ there have been no studies evaluating the disease modeling capacity of these cells in comparison to their primed counterparts.

We previously identified DNA methylation changes associated with abnormal trophoblast differentiation in PE-affected iPSC.^32^ Here, we expand on our earlier findings by utilizing isogenic naïve- and primed iPSC-derived TSC, the former of which undergoes dramatic loss of DNA methylation during naïve conversion, in order to probe the epigenetic mechanisms of PE pathogenesis. We applied an improved EVT differentiation protocol to our control and PE-affected naïve and primed iPSC-derived TSC, and compared their detailed cellular phenotype, global gene expression, and DNA methylation changes to elucidate the pathogenesis of PE.

## Results

### TSC derivation of PE- and control-iPSC from both primed and naïve state pluripotent stem cells

**Figure 1:**
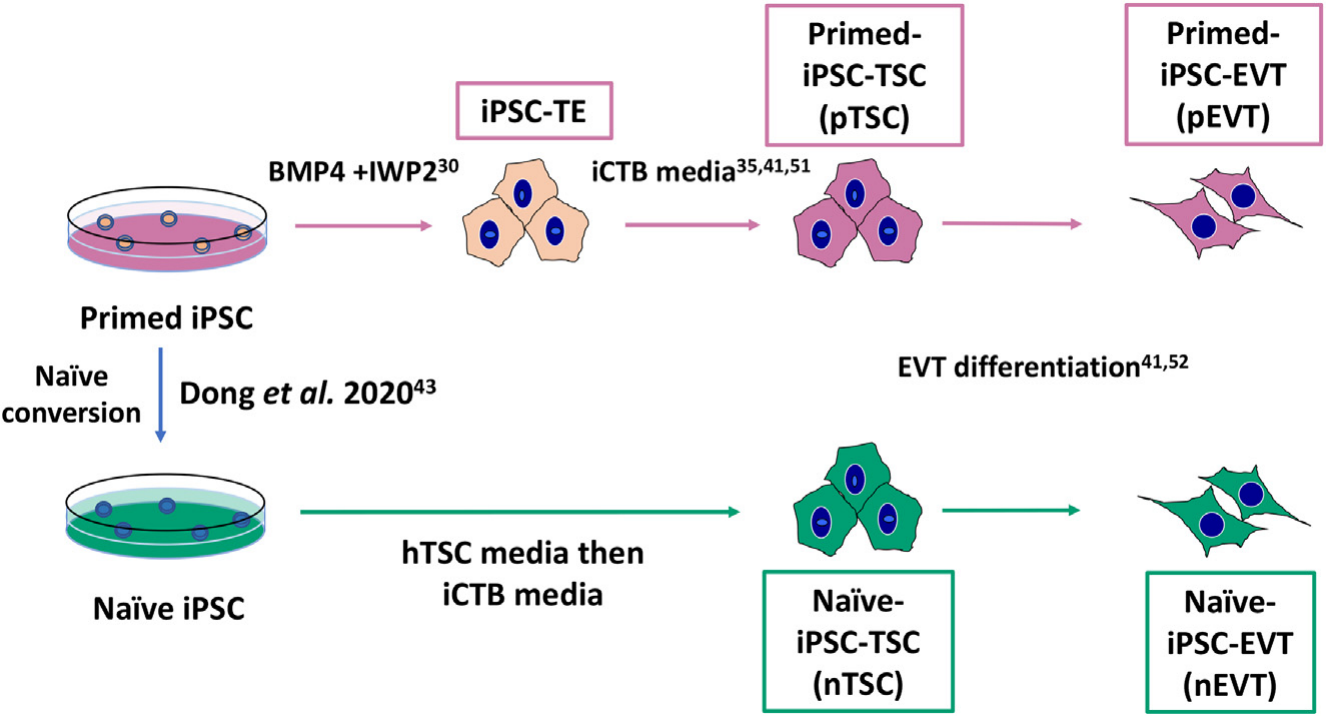
Schematics of experimental design Schematics of experimental design for deriving “naïve”- and “primed”-TSC lines, and EVT differentiation. TE: trophectoderm. References are listed for specific publications at each step.

We used three PE- and three control-iPSC lines that were reprogrammed and characterized previously.^32^ Primed iPSC-derived TSC were generated using a recently established protocol.^35^ Trophectoderm (TE) induction was first initiated by 4 days of treatment with BMP4 and WNT inhibitor IWP2.^30^ The purity of the culture was verified using the surface expression of EGFR, a CTB marker, by flow cytometry. Over 90% of cells were EGFR^+^, replated into a modified TSC medium, referred to as iCTB medium,^35^^,^^51^ and passaged 4–5 times, in order to establish primed iPSC-derived TSC (pTSC).^35^ The same set of iPSC lines were used to derive naïve iPSC-TSC (nTSC), by first converting the iPSC to a naïve state, then culturing in TSC media for 5 passages to establish nTSC (Figure 1).^43^ One control iPSC line (1938) failed to convert to nTSC. We therefore instead used the AN1 naïve iPSC nTSC line derived by Dong et al. (2020) as a control, which was previously characterized and is not known to have a history of pregnancy complications.^43^ Gene expression of PSC markers and global methylation levels of our primed- and naïve-iPSC were checked by RNA-seq and whole genome bisulfite sequencing (WGBS). We confirmed that our primed- and naïve-iPSC lines show cell-type-specific marker expression and loss of DNA methylation during naïve conversion. As expected, our primed iPSC lines expressed the primed PSC markers ZIC2 and SFRP2 and were hypermethylated (over 80%), compared to our naïve-iPSC, which expressed the naïve PSC markers KLF17 and DNMT3L and were hypomethylated (about 40%) (Figures S1A and S1B). These data confirm that our primed-to-naïve conversion was successful, and that the DNA methylation levels of our lines are similar to previously reported human naïve iPSC.^50^

TSC derivation from both primed and naïve iPSC was confirmed by a combination of gene expression, methylation, and through an *in vivo* tumor formation assay. Principal component analysis (PCA) using gene expression data via RNA-seq, confirmed that both naïve and primed TSC are similar to previously reported primary TSCs (Figure S1C).^52^ Using DEseq2 normalized gene expression, we calculated the Euclidian distance between our samples and found that both naïve- and primed-iPSC-derived TSC cluster together with the primary TSC (Figure S1D). These data show that, consistent with previous findings, naïve- and primed-iPSC derived TSC are transcriptionally similar to primary TSCs.^35^^,^^43^^,^^45^^,^^46^ We further verified that the promoter region of *ELF5* was hypomethylated in our iPSC-derived TSC as has been described (Figure S1E).^53^ Lastly, we tested the tumor formation potential of our TSC *in vivo* and found that both our naïve and primed hTSC lines can form trophoblastic tumors when injected into NOD-SCID mice (Figure S1F).

In summary, both our naïve and primed iPSC-derived TSC show trophoblast characteristics, including gene expression profiles similar to primary TSC, *ELF5* promoter hypomethylation, and the ability to form *in vivo* trophoblastic tumors, consistent with previous reports.^35^^,^^37^^,^^45^^,^^46^ These 12 TSC lines (3 control and 3 PE naïve and primed iPSC-derived TSC) were used for subsequent cellular assays, gene expression profiling, and DNA methylation analyses.

### Characterization of TSC from PE and control iPSC

**Figure 2:**
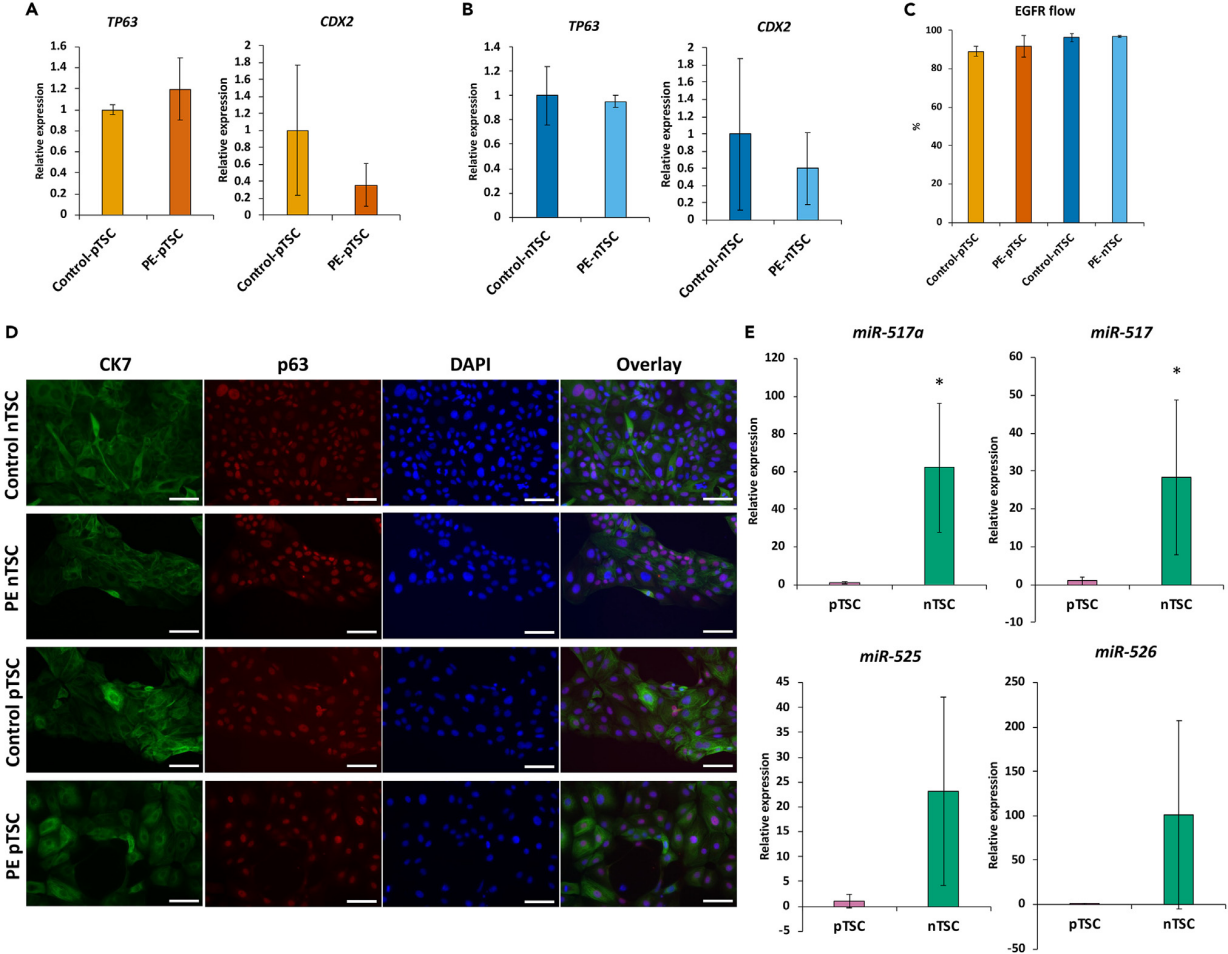
Characterization of TSC from PE and control iPSC Bar graph displaying cellular analysis of TSC state. qPCR of CTB markers *TP63* and *CDX2* in (A) PE-pTSC compared to control-pTSC, and (B) PE-nTSC compared to control-nTSC, normalized to L19 and shown as fold change over control TSC. (C) Flow cytometric analysis of control-pTSC, PE-pTSC, control-nTSC, and PE-nTSC for CTB marker EGFR as percent expression. (D) Immunocytochemistry on iPSC-derived TSCs stained with TSC markers p63 and CK7. Scale bar 75μm. (E) Bar graphs displaying qPCR of selected C19MC genes, normalized to hsa-miR-103a-3p and shown as fold change over pTSC. Bar graph display mean ± standard deviation of triplicates. ∗p < 0.05.

Our initial study using these 3 PE and 3 control iPSC lines differentiated to TE by 4 days treatment with BMP4+IWP2 showed no functional, molecular, or morphological differences between PE and control lines at this stage.^32^ We similarly compared our control and PE iPSC-derived TSC and found that CTB markers *CDX2* and *TP63* showed similar transcript expression levels using RT-qPCR (qPCR) (Figures 2A, 2B; S2A). Additionally, flow cytometric analysis showed that surface expression of EGFR was consistently over 80% in both PE and control TSC (Figure 2C). Immunofluorescence analysis showed the protein expression of TSC markers, p63 and CK7, was similar between the PE and control TSC (Figure 2D). Next, we performed PCA analysis using iPSC-derived TSC RNA-seq data, and found that the component with the largest amount of variance (PC1: 22%) differentiated between the derivation method of the TSC (i.e., whether they were sourced from naïve or primed iPSC), while differences between PE and control iPSC-derived TSC did not appear until PC5 (6%) (Figure S2B). We also compared a list of differentially expressed genes (DEGs) generated by comparing our TSC lines and their respective iPSC state, to a previously published list of primary CTB-specific genes,^42^ and found that our control-pTSC, PE-pTSC, control-nTSC, and PE-nTSC showed over 70% similarity across the four groups, suggesting our control and PE TSC lines are transcriptionally similar (Table S1). In addition, hierarchical clustering using Euclidean distance showed that our control and PE TSC lines clustered together, suggesting that at the TSC cell state, our lines do not show obvious transcriptomic differences due to their disease state or iPSC state (see Figure S1D). Likewise, hierarchical clustering and correlation analysis using our WGBS data showed that our PE and control TSC lines of naïve and primed origin were similar to primary TSC lines compared to their iPSC or mesenchymal stem cells (MSCs) (Figure S2C).

We next investigated cell proliferation and the expression of chromosome 19 miRNA cluster (C19MC) in our TSC, as these marks were recently reported to be different between nTSC and pTSC.^46^ We found that, consistent with Kobayashi et al. (2022), nTSC showed higher cell proliferation compared to pTSC; however, there were no differences between our control and PE disease state (Figure S2D). We also found that miR-517 and miR-517a were expressed at significantly higher levels in our nTSC compared to our pTSC, and likewise miR-525 and miR-526 also trended higher in nTSC. We therefore concluded that C19MC expression is increased in nTSC compared to pTSC (Figures 2E; S2E). Moreover, the expression of these four miRNAs was correlated with C19MC class 1 promoter methylation, which was hypomethylated in nTSC compared to pTSC (Figure S2F).^46^ In summary, we found that, compared to control iPSC, TE induction and TSC derivation are not compromised in PE-iPSC based on expression of specific CTB-associated markers and whole transcriptome analysis, but there are notable differences at the C19MC region between our primed and naïve iPSC-derived TSC at the DNA methylation and expression levels.

### Primed iPSC-derived PE EVT show blunted EVT formation and function

Since primary TSC derivation and lineage-specific differentiation were first reported,^42^ iPSC-derived trophoblast differentiation protocols have improved significantly. In our previous work,^32^ EVT differentiation was sub-optimal and resulted in a heterogeneous culture of differentiated derivatives. In this study, conversion of control and PE iPSC to TSC allowed for lineage-specific differentiation into a pure EVT population, using an updated differentiation protocol.^35^^,^^41^^,^^52^

**Figure 3:**
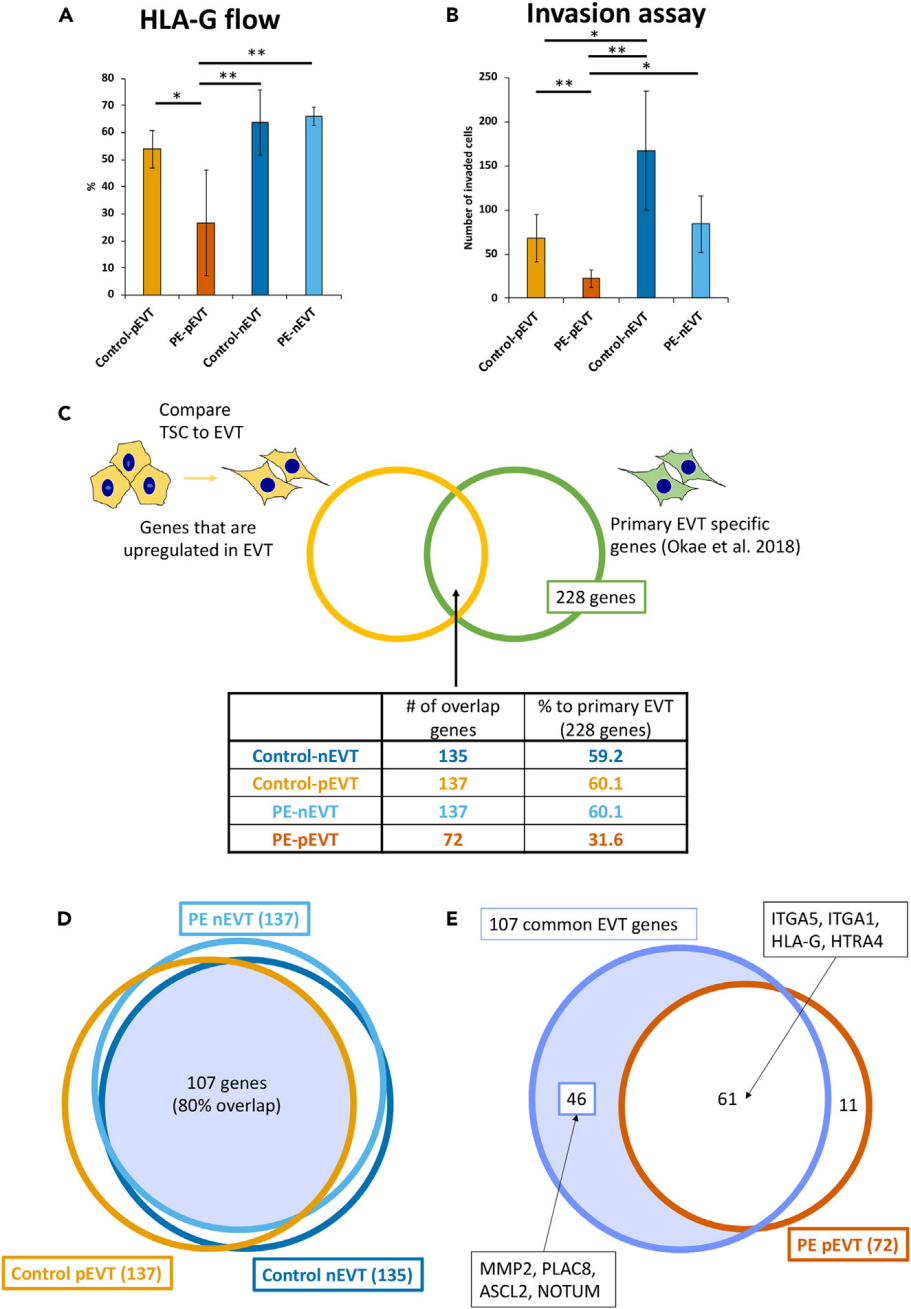
Primed iPSC-derived PE EVT show blunted EVT formation and function Bar graph displaying cellular analysis of EVT state. (A) Flow cytometric analysis of Control-pEVT, PE-pEVT, control-nEVT, and PE-nEVT for EVT marker HLA-G as percent expression. (B) Number of invaded cells by Matrigel invasion assay. (C) Table showing number of differentially expressed genes upregulated in EVT state compared to its respective TSC state, and contrasted to EVT specific genes (Okae et al. 2018; *n* = 228 genes). (D) Venn diagram displaying the 107 EVT-specific genes overlapping in control-pEVT (*n* = 137), control-nEVT(*n* = 135), and PE-nEVT (*n* = 137) from Figure 3C. (E) Venn diagram displaying overlap between 107 common EVT genes (from Figure 3D) and PE-pEVT (*n* = 72) from Figure 3C. Bar graph display mean ± standard deviation of triplicates. ∗p < 0.05, ∗∗<0.01.

Following EVT differentiation, we found that PE-affected primed iPSC-derived EVT (PE-pEVT) showed significantly lower expression of the EVT marker, HLA-G, by flow cytometry, compared to control primed iPSC-derived EVT (control-pEVT), as well as both control and PE-affected naïve iPSC-derived EVT (control- and PE-nEVT) (Figure 3A). Additionally, we assessed the invasive ability of these EVT, using a Matrigel invasion assay and found that PE-pEVT showed significantly lower invasion compared to control-pEVT, and both control- and PE-nEVT (Figure 3B).

We next compared how well our lines differentiated into EVT by investigating the DEG that are upregulated in EVT compared to their respective TSC state, as well as to a previously published primary EVT gene set.^42^ PE-pEVT showed upregulation of only 31.6% of primary EVT-specific genes, while all other EVT (control-pEVT, control-nEVT, and PE-nEVT) expressed about 60% of the same EVT-specific genes (Figures 3C; Table S2). These data suggest that differentiated PE-pEVT are less similar to primary EVT, compared to control-pEVT, control-nEVT, and PE-nEVT. Next, we compared EVT-specific genes upregulated in control-pEVT (137 genes), control-nEVT (135 genes), and PE-nEVT (137 genes), compared to the TSC state (see Figure 3C), and found that almost 80% (107 genes of 135–137 genes) of these genes were shared between the three conditions (Figures 3D; Table S3). We next compared these 107 common EVT genes (from Figure 3D) to the differentially upregulated genes specific to PE-pEVT (72 genes; see Figure 3C), and found that only 45% (61 genes of 135–137 genes), including the EVT markers *ITGA5*, *HLA-G*, and *HTRA4*, were also upregulated in PE-pEVT (Figure 3E). These data suggest that PE-associated defects in EVT formation were limited to PE-pEVT, and were lost when iPSCs were converted to the naïve state and differentiated into nEVT.

Gene ontology (GO) analysis of the 46 EVT genes, including the EVT markers *MMP2*, *PLAC8*, *ASCL2*, and *NOTUM*, which were not significantly upregulated in the PE-pEVT compared to the PE-pTSC state (Figures 3E; S3A), showed that PE-pEVT are deficient in extracellular matrix (ECM)-related pathways (Figure S3B).

We also investigated PE-pEVT-specific genes at the EVT state. To extract these genes, we identified the overlapping DEGs from the comparison of PE-pEVT vs. PE-nEVT, and from PE-pEVT vs. control-pEVT, and found 344 genes that were upregulated, and 665 genes that were downregulated specifically in PE-pEVT (Figure S3C; Table S4). The 665 downregulated genes specific to PE-pEVT included EVT-associated genes, such as *ITGA5*, *ITGA1*, *ASCL2*, *PLAC8*, *MMP2*, and *NOTUM*. We used PlacentaCellEnrich^54^ to confirm that these 665 downregulated genes were significantly associated with EVT, whereas the 344 genes specifically upregulated in PE-pEVT did not show any significant association with a specific placental cell type (Figure S3C). Gene ontology analysis of the 665 genes downregulated in PE-pEVT showed enrichment in protein glycosylation and ECM-associated pathways (Figure S3D).

In summary, cellular and transcriptomic data analyses showed impaired EVT differentiation/function, exclusively in PE-pEVT. We found that PE-pEVT had the least similarity to a previously published primary EVT gene expression signature, and this phenotype was erased following naïve conversion (PE-nEVT), suggesting that abnormal EVT differentiation/function may be regulated through epigenetic mechanisms.

### DNA methylation influences phenotypic differences between PE and control iPSC-derived trophoblast

Our cellular and transcriptomic analyses indicated that our naïve converted PE-TSC did not harbor defects in EVT formation, suggesting that loss of epigenetic marks during naïve conversion might be responsible for the blunted EVT formation phenotype seen in our PE-pEVT. Therefore, we performed WGBS of our PE- and control-iPSC-derived TSCs from both the naïve and primed state.

**Figure 4:**
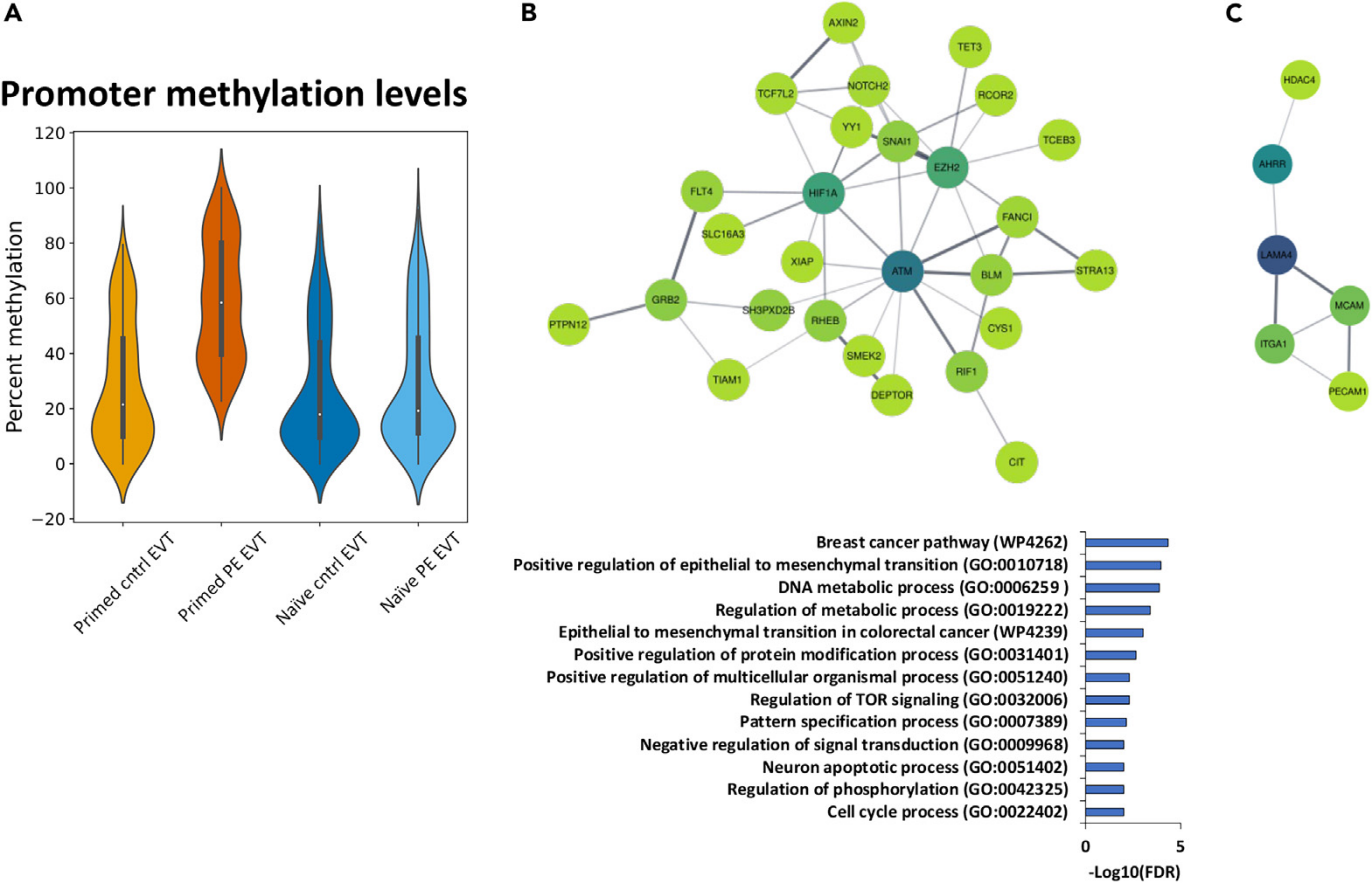
DNA methylation influences phenotypic differences between PE and Control iPSC-derived trophoblast (A) Violin plot displaying 321 genes that are specifically downregulated in PE-pEVT, compared to three other conditions. (B) Largest cluster from the PPI network analysis showing hub genes specific to 321 genes. Significant pathways identified from the PPI network analysis are shown in the table. (C) Smaller cluster from the PPI network analysis showing hub genes specific to 321 genes.

We focused our analysis on the 665 transcriptionally downregulated genes specific to our PE-pEVT (see Figure S3C right panel), and investigated the % DNA methylation of active promoter regions marked by tri-methylation of histone H3 at lysine 4 (H3K4me3) and DNase hypersensitivity from the Encyclopedia of DNA Elements (ENCODE).^55^^,^^56^ There were 321 differentially hypermethylated (at least 20% methylation difference) promoter regions in PE-pEVT compared to control-pEVT, control-nEVT, and PE-nEVT (Figure 4A). We analyzed the genes regulated by these 321 differentially methylated promoter regions using the STRING protein-protein interaction (PPI) database through Cytoscape.^57^^,^^58^ Following network creation and clustering, GO analysis revealed 13 pathways in the largest cluster (Figure 4B), and 1 pathway from a smaller cluster (Figure 4C) that were statistically significant. Interestingly, the genes downregulated in PE-pEVT were enriched for pathways such as ECM organization and positive regulation of epithelial mesenchymal transition (EMT) (Figure 4B). PPI analysis also pointed to miRNA targets in ECM and membrane receptors (WP2911, FDR = 0.0146) from the smaller cluster (Figure 4C). Notably, for the largest PPI cluster, three of the hub genes consisted of the EVT invasion-associated gene hypoxia-inducible factor 1-alpha (HIF1A),^59^ the trophoblast proliferation and migration gene enhancer of zeste homolog 2 (EZH2),^60^ which is a histone methyltransferase that catalyzes histone H3 on lysine 27 (H3K27) for silencing transcription,^61^ and the cell growth and DNA damage repair gene ataxia-telangiesctasia mutated (ATM).^62^ We also found other genes associated with cell migration/invasion, histone modification, and DNA methylation that were not identified as hub genes in the PPI analysis. These genes include histone deacetylase 4 (HDAC4), ten-eleven translocation methylcytosine dioxygenase 1 (TET1), ten-eleven translocation methylcytosine dioxygenase 3 (TET3), placenta specific protein 8 (PLAC8), and ubiquitin associated and SH3 domain containing B (UBASH3B) (Figure S4). These results indicate that our PE-pEVT have both significant downregulation of gene expression and promoter hypermethylation of cell invasion-associated genes and histone and DNA methylation modification enzymes, suggesting epigenetic regulation is altered in PE pathogenesis.

We also examined the % DNA methylation of active promoter regions marked by H3K4me3 and DNase hypersensitivity in the 344 genes that were differentially upregulated in PE-pEVT (see Figure S3C, left panel). We found that there were 137 genes that were hypomethylated at the active promoter region in PE-pEVT compared to control-pEVT (Figure S5A), but only 18 genes remained that were statistically upregulated in gene expression and hypomethylated (at least 20% methylation difference) when the comparison was expanded to all three groups (control-pEVT, control-nEVT, and PE-nEVT) (Table S5). PPI and GO analysis of these 137 genes showed negative regulation of vascular endothelial growth factor (VEGF) receptor signaling pathway (Figure S5B). GO analysis on the 18 genes upregulated and containing promoter hypomethylation were still statistically enriched for the VEGF receptor signaling pathway (GO:0030947; Adjusted *p* value <0.05). Genes associated with this pathway were growth factor receptor bound protein 10 (GRB10), a known maternally expressed imprinted gene in the placenta,^63^^,^^64^ and the tumor angiogenesis linked gene Multimerin 2 (MMRN2), which is known to be degraded by MMP9 (Figure S5C).^65^

In conclusion, these analyses, along with our cellular phenotypic characterization, indicate that methylation differences between our primed PE and control iPSC models may explain why abnormal EVT differentiation and function were exclusively observed in EVT derived from primed, but not naïve, iPSC.

## Discussion

Comparatively little is known about PE, in part due to the limitations of animal models for placenta-based disease, and lack of (up-to-now) a tractable *in vitro* model system for the disease. An iPSC-based model not only allows for the study of PE pathogenesis, but also enables exploration and manipulation of the genetic or epigenetic makeup of the model. This is particularly important when studying placenta-based pregnancy disorders as they are known to be epigenetically affected by maternal lifestyle and the uterine environment.^38^^,^^39^^,^^40^ Further, iPSC-based models have the added benefit of known patient information, including placental pathology and pregnancy outcome. We therefore developed an iPSC-based model system to elucidate the causes of PE, and to test future therapies.

In our previous study,^32^ we characterized the PE cellular phenotype associated with MVM using an iPSC-based model system, and reported that the PE-iPSC-derived trophoblast had a blunted response to changes in oxygen tension. Gene expression data of PE-iPSC-derived trophoblast showed similarities to that of PE placenta, and pointed to pathways through which trophoblast derived from PE-iPSC were more susceptible to environmental stressors. Analysis of DNA methylation data also identified alterations in promoter regions of genes involved in response to oxygen tension, further explaining these cellular phenotypes.^32^ However, the EVT differentiation protocol used in this previous study was sub-optimal.^41^ Technological advancements in *in vitro* hTSC derivation, culture, and differentiation^35^^,^^41^^,^^42^^,^^52^ have enabled us to convert iPSC to TSC, and thus better model EVT differentiation in the current study. We therefore performed in-depth characterization of EVT, differentiated from naïve and primed iPSC-derived TSC, from the same placenta-derived iPSC as previously described.^32^

We first compared PE- and control-TSC and found that, consistent with our previous findings,^32^ TSC induction was not compromised. Differences between PE- and control-derived trophoblasts became clear only when the cells were further differentiated into EVT. Compared to control-pTSC, PE-associated pTSC show abnormal EVT differentiation, characterized by reduced HLA-G surface expression and invasive capacity. RNA-seq analysis revealed that our PE-pTSC differentiation into EVT only upregulated ∼30% (72 genes) of EVT-specific genes, compared to the other three conditions which all upregulated ∼60% (135–137 genes). GO analysis of the genes that are significantly downregulated in PE-pEVT identified enrichment in protein glycosylation and ECM-associated pathways, all of which are known for abnormalities associated with PE.^66^^,^^67^^,^^68^^,^^69^ This suggests that abnormalities in PE-pEVT include a lack of ECM remodeling, and therefore led to deficient invasive capacity. Furthermore, our isogenic PE-nEVT lines did not show similar differentiation defects to PE-pEVT, but exhibited surface expression of EVT marker HLA-G, expression of EVT-specific genes, and invasion functions similar to control lines. These data strongly suggest that EVT invasive capacity is regulated through epigenetic mechanisms. Thus, we next performed WGBS on these cells to identify PE-associated epigenetic changes in PE-pEVT. Global differential methylation analysis was difficult due to our small sample size and inherent differences between methylation levels in naïve- and primed-derived cell lines, therefore, we focused on differential methylation levels in our DEG. We found that differential hypermethylation in promoter regions of PE-pEVT was enriched in pathways associated with ECM organization and positive regulation of EMT. ECM-associated pathways have previously been shown to be associated with PE-EVT defects using placental tissues and mouse models.^66^^,^^67^^,^^68^ These data support the poor invasion seen in our PE-pEVT, and suggests that hypermethylation at the promoter region of EMT-associated genes leads to deficient ECM organization, which is necessary to be able to properly invade the myometrium.

Interestingly, we also found numerous genes in PE-pEVT with downregulation in gene expression and hypermethylation at their promoter region that are associated with trophoblast cell invasion and ECM organization. These genes include *HIF1A*, *PLAC8*, and *UBASH3B*, all of which are associated with trophoblast cell invasion and ECM organization. Among these genes, we noted discrepancies between our *HIF1A* results and the literature. *HIF1A* is reported to be increased in preeclamptic placenta,^70^^,^^71^ while our data showed reduced gene expression. We postulate that these discrepancies may emanate from the different time points of *HIF1A* measurements. Our EVT differentiation model is capturing the differentiation of CTB to EVT, which occurs before the 8^th^ week of gestation,^72^ while studies of PE placental villous explants *HIF1A* expression are from a much later time point during the second trimester of pregnancy. Because PE placenta is exposed to chronic hypoxia due to the lack of spiral artery remodeling in early gestation, it is possible that an accumulation of placental hypoxia may affect *HIF1A* expression in later gestation.^70^^,^^71^ Additionally, most gene expression information from PE placentas include mixture of cell types, whereas our model system allows us to measure gene expression from pure EVT specifically. Other interesting genes that were downregulated in gene expression and hypermethylated at their promoter were those associated with histone modification and DNA methylation. Epigenetic modifications might in part explain why placental tissues from patients with PE have been reported to have more downregulated genes than their unaffected controls.^73^ *EZH2* is a histone methyltransferase that catalyzes H3K27 to silence transcription,^61^ and is reduced in PE placenta.^60^ *EZH2* is also known to promote EMT by epigenetically silencing CDX1 which then represses MMP9 by directly binding to its promoter region, resulting in trimethylation of H3K27.^74^^,^^75^ Another chromatin modifier dysregulated in our data are *HDAC4*. *HDAC4* is a part of the HDAC class IIa family, which catalyzes and removes acetylation from lysine residues and loosens compact chromatin structures. This, in turn, allows RNA polymerase to initiate transcription, resulting in a decrease in gene repression at target gene promoters.^76^^,^^77^ *HDAC4* is reported to be downregulated in PE placenta,^78^ stimulates vascular smooth muscle cell migration,^79^ and promotes cancer cell invasion.^80^ Finally, TET enzymes are known to catalyze the DNA demethylation process by converting 5-methylcytosine (5mC) to 5-hydroxymethylcytosine (5hmC), and then further converting 5hmC into 5-formylcytosine (5fC) and 5-carboxylcytosine (5caC).^81^^,^^82^^,^^83^ Both *TET1* and *TET3* are reported to be downregulated in PE placenta, indicating an important role in PE pathogenesis.^84^ Our results suggest that promoter hypermethylation of genes important to cell migration and invasion, as well as DNA methylation and histone modifiers are involved in PE pathogenesis. Surprisingly, despite these promoter methylation differences in key EMT and DNA methylation/histone modification-associated genes, our examination of 46 EVT-specific genes that show reduced expression in PE-pEVT (see Figure 3E) did not show correlation with promoter hypermethylation, suggesting that these genes might be regulated by other epigenetic mechanisms, such as chromatin modification or accessibility. Overall, we have demonstrated that preservation of a PE epigenetic landscape is required for modeling the PE-specific phenotype.

Kobayashi et al. (2022) showed that miRNAs located in the C19MC show significant gene expression differences between naïve and primed hPSC-derived TSC, due to promoter methylation differences, and that C19MC expression level is important for cell proliferation and differentiation capacity of hTSC.^46^ Consistent with their report, our pTSC showed class 1 promoter hypermethylation at C19MC, low chromosome 19 microRNA expression (in both control and PE-associated lines), low cell proliferation, and blunted terminal trophoblast differentiation, when compared to nTSC. Additionally, we confirmed that nTSC has consistently higher proliferation compared to pTSC, but surprisingly we did not find differences in cell proliferation between PE-affected and control TSC derived from either naïve or primed iPSC. We were able to successfully derive primed iPSC and differentiate them into EVT with similar gene expression and functional characteristics to naïve iPSC-derived EVT. However, abnormalities in EVT differentiation were exclusively seen using PE-affected pTSC, despite similar C19MC expression levels to control pTSC lines. Therefore, our data suggest that C19MC is not responsible for the blunted EVT differentiation phenotype seen in our PE-pEVT.

### Limitations of the study

The limitation of our study is that we focused on DNA methylation, and did not investigate differences in chromatin accessibility or modifications. Additionally, consistent with previous studies,^35^^,^^45^^,^^46^ we also noted primed-iPSC-derived TSC lack C19MC expression. Finally, we were unable to convert one of our control (1938) lines to a naïve state. As a result, we included the well-characterized AN1 naïve iPSC-derived TSC line^43^ in our analysis. We note that the addition or removal of this cell line did not change the overall results of our PE-associated EVT defect.

### Conclusion

In conclusion, we have leveraged recently developed protocols for deriving TSC from naïve and primed iPSC to compare the molecular and functional properties of specialized trophoblast cells from control and PE patients. Our study demonstrates that primed-iPSC-derived TSC and their EVT derivatives retain epigenetic memory of the PE placenta and show a lack of EMT-associated gene expression. These phenotypes correlate with promoter hypermethylation, which we suggest might be a possible underlying mechanism of this disease. Thus, our study demonstrates the value of comparing trophoblast cells derived from isogenic naïve and primed iPSC to uncover a potential epigenetic component in PE pathogenesis. Although our work focuses on modeling trophoblast in PE placenta with MVM, the utility of our model could be expanded to different types of placental injuries and cell types. This system will offer researchers a tool for the future development of biomarker screening and the potential development of therapeutics for reversing the PE disease phenotype.

## STAR★Methods

### Key resources table

**Table:** 

REAGENT or RESOURCE	SOURCE	IDENTIFIER
**Antibodies**		

APC conjugated mouse anti-human EGFR Antibody (clone AY13)	BioLegend	BioLegend Cat# 352906; RRID: AB_11150410
APC mouse IgG1	BioLegend	BioLegend Cat# 400122; RRID: AB_326443
PE conjugated mouse anti-HLA-G (clone MEM-G9)	EXBIO	EXBIO Praha Cat# 1P-292_C100; RRID: AB_10736086
PE mouse IgG1	BioLegend	BioLegend Cat# 400112; RRID: AB_2847829
mouse anti-p40 (delta-N isoform of TP63)	Biocare Medical	Biocare Medical Cat# ACI 3066 C; RRID: AB_2858274
rabbit anti-KRT7 antibody	Abcam	Abcam Cat# ab68459; RRID: AB_1139824
DAPI	Invitrogen	Invitrogen Cat# D1306; RRID: AB_2629482

**Chemicals, peptides, and recombinant proteins**		

Advanced DMEM/F-12	ThermoFisher	12634028
A 83-01	Tocris	TB2939-RMU
Bone morphogenetic protein 4	R&D Systems	314-BP
Bovine Serum Albumin solution (30%)	Gemini Bio-Products	700-110-100
CHIR99021	MilliporeSigma	SML1046
Collagen IV	MilliporeSigma	C0543
4′,6 Diamidino 2 Phenylindole, Dihydrochloride	ThermoFisher	D1306
DMEM/F12, HEPES	ThermoFisher	11330–032
DMEM/F-12 no HEPES	ThermoFisher	11320–033
EDTA	ThermoFisher	15575020
Geltrex, growth factor-reduced	ThermoFisher	A1413302
Insulin-transferrin-sodium selenite	MilliporeSigma	I1884
ITS-X	ThermoFisher	51500056
IWP-2	Selleck Chemicals	S7085
Knockout serum replacement	ThermoFisher	10828028
L-ascorbic acid 2-phosphate	MilliporeSigma	A8960
Matrigel, Basement Membrane Matrix, LDEV-free	Corning	354234
2-Mercaptoethanol	ThermoFisher	21985-023
NRG1	Abcam	ab50227
Recombinant human epidermal growth factor	R&D Systems	236-EG
Recombinant human basic fibroblast growth factor 2	BioPioneer	HRP-0011
Recombinant human hepatocyte growth factor	STEMCELL Technologies	78019.2
SB431542	MilliporeSigma	616464
TryPLE Express	ThermoFisher	12604021
Valproic acid sodium salt	MilliporeSigma	676380
Y-27632	Selleck Chemicals	S1049

**Critical commercial assays**		

*mir*Vana miRNA Isolation Kit	ThermoFisher	AM1561
Qubit RNA BR assay	ThermoFisher	Q10210
TruSeq Stranded mRNA Library Prep	illumina	20020595
DNeasy	Qiagen	69506
Qubit dsDNA BR assay	ThermoFisher	Q32853
Accel-NGS Methyl-seq kit	Swift Biosciences	30096
Click-iT EdU Alexa Fluor 488 Flow Cytometry Assay Kit	ThermoFisher	C10425
TaqMan Advanced miRNA cDNA Synthesis Kit	ThermoFisher	A28007
Applied Biosystems TaqMan Fast Advance Master Mix	ThermoFisher	4444556
Taqman probe-hsa-miR-517a-3p	ThermoFisher	A25567-479485_mir
Taqman probe-hsa-miR-517-5p	ThermoFisher	A25567-478980_mir
Taqman probe-hsa-miR-525-3p	ThermoFisher	A25567-478995_mir
Taqman probe-hsa-miR-526b-3p	ThermoFisher	A25567-478996_mir
Taqman probe-miR-103a-3p	ThermoFisher	A25567-478253_mir

**Deposited data**		

RNA-seq data	this paper	GSE243579; https://www.ncbi.nlm.nih.gov/geo/query/acc.cgi?acc=GSE243579
WGBS data	this paper	SRA under the BioProject PRJNA1019896; https://dataview.ncbi.nlm.nih.gov/object/PRJNA1019896?reviewer=lv96435b448o06c98f1dh8bvd

**Experimental models: Cell lines**		

Human: 1754 iPS3	Horii et al. ^32^	N/A
Human: 1932 iPS2	Horii et al. ^32^	N/A
Human: 1933 iPS5	Horii et al. ^32^	N/A
Human: 1938 iPS3	Horii et al. ^32^	N/A
Human: 1947 iPS8	Horii et al. ^32^	N/A
Human: 1981 iPS11	Horii et al. ^32^	N/A
AN1 naïve iPSC nTSC	Dong et al. ^32^	N/A

**Experimental models: Organisms/strains**		

Mouse: NOD.Cg-*Prkdc*^*scid*^ *Il2rg*^*tm1Wjl*^/SzJ	The Jackson Laboratory	JAX: 005557

**Oligonucleotides**		

ΔNp63 isoform primer	IDT	F 5’-CTG GAA AAC AAT GCC CAG A -3’ R 5’-AGA GAG CAT CGA AGG TGG AG -3’
CDX2 primer	IDT	F 5’-TTC ACT ACA GTC GCT ACA TCA CC -3’ R 5’-TTG ATT TTC CTC TCC TTT GCT C -3’
L19 primer	IDT	F 5’ -AAA ACA AGC GGA TTC TCA TGG A- 3’ R 5’ -TGC GTG CTT CCT TGG TCT TAG- 3’

**Software and algorithms**		

BiomaRt	Durinck et al. ^85^	https://bioconductor.org/packages/release/bioc/html/biomaRt.html
Bismark	Krueger F et al. ^86^	https://github.com/FelixKrueger/Bismark
Bowtie2	Langmead et al. ^87^	https://github.com/BenLangmead/bowtie2
CpGtools	Wei et al. ^56^	https://cpgtools.readthedocs.io/en/latest/overview.html
Cutadapt	Martin M. ^88^	https://github.com/marcelm/cutadapt
Cytoscape	Shannon et al. ^58^	https://cytoscape.org
DESeq2	Love et al. ^89^	https://bioconductor.org/packages/release/bioc/html/DESeq2.html
Fastq Screen	Wingett et al. ^90^	https://www.bioinformatics.babraham.ac.uk/projects/fastq_screen/_build/html/index.html
featureCounts	Liao et al. ^91^	https://subread.sourceforge.net/featureCounts.html
FGSEA	Korotkevich et al. 2019	https://bioconductor.org/packages/release/bioc/html/fgsea.html
ggplot2	Wickham et al. 2016	https://cran.r-project.org/web/packages/ggplot2/index.html
ImageJ	Schneider et al. ^92^	https://imagej.nih.gov/ij/
lmerTest	Kuznetsova et al. ^93^	https://cran.r-project.org/web/packages/lmerTest/
lqmm	Geraci M. ^94^	https://cran.r-project.org/web/packages/lqmm/index.html
multiQC	Ewels et al. ^95^	https://multiqc.info/docs/
Qlucore Omics Explorer	Qlucore	https://qlucore.com
R	R Core Team. 2018	https://www.r-project.org
STAR	Dobin et al. ^96^	https://github.com/alexdobin/STAR
TrimGalore	The Babraham Institute	https://github.com/FelixKrueger/TrimGalore
Cytoscape	Shannon et al. ^58^	https://cytoscape.org

### Resource availability

#### Lead contact

Further information and requests for resources and reagents should be directed to and will be fulfilled by the lead contact, Mariko Horii (mhorii@health.ucsd.edu).

#### Materials availability

This study did not generate new unique reagents.

#### Data and code availability

• RNA-seq data have been deposited to the Gene Expression Omnibus: GSE243579. WGBS data are deposited to SRA under the BioProject: PRJNA1019896. These data are publicly available as of the date of publication. Accession numbers are also listed in the key resources table.
• This paper does not report original code.
• Any additional information required to reanalyze the data reported in this paper is available from the lead contact.

### Experimental model and study participant details

#### Generation of primed- and naïve- TSC lines from primed PSC

Derivation of the TSC line from primed hPSC was performed under a protocol approved by the UCSD Institutional Review Board and Embryonic Stem Cell Research Oversight Committee (ESCRO Protocol number: 171648). To protect donor privacy and confidentiality, all samples were coded and de-identified in this study. Previously established and characterized primed-iPSCs, which preeclampsia (PE) with severe features showed placental pathologic findings of MVM, and control (non-PE) cases show absence of maternal hypertensive disease or fetal growth restriction, with no evidence of MVM or other placental disc lesions, were used in the study.^32^^,^^43^ pTSC was derived using the previously published protocol.^35^^,^^41^ In brief, primed-iPSC were cultured on Geltrex- (ThermoFisher) coated plates (using 1:200 diluted Geltrex) in StemFlex (ThermoFisher) or mTeSR Plus (Stem Cell Technologies). The cells were differentiated to trophectoderm (TE)-like cells using the first step of a previously established protocol.^30^ In brief, hPSCs were dissociated using TrypLE Express (ThermoFisher) and plated for 1.0 x10^5^ cells/well of the 6 well plate coated with Geltrex (ThermoFisher) in the presence of 5μM Y-27632 (Selleck Chemicals). The next day, media was changed to first-step differentiation media: DMEM/F12 (ThermoFisher), with 1x ITS (Millipore-Sigma), 64μg/ml L-ascorbic acid (Millipore-Sigma), 543μg/ml NaHCO3 (Fisher Scientific), 2% BSA (Gemini), 10 ng/mL BMP4 (ThermoFisher), and 2μM IWP2 (Selleck Chemicals). Media was changed every day for 4 days. Conversion into TE-like cells was confirmed based on surface EGFR expression of over 90% by flow cytometry. Subsequently, cells were replated on collagen IV (Millipore Sigma, 5 μg/mL) in iCTB media: Advanced DMEM/F12 (ThermoFisher), 1X N2 (ThermoFisher), 1X B27 (ThermoFisher), 1X Glutmax (ThermoFisher), 150 μM 1-thioglycerol (Millipore Sigma), 0.05% BSA (Gemini), 1% knockout serum replacement (KSR; ThermoFisher), 2 μM CHIR99021 (Millipore Sigma), 0.5 μM A83-01 (Tocris), 1 μM SB431542 (Millipore Sigma), 5 μM Y27632 (Selleck Chemicals), 130 μg/mL VPA sodium salt (Millipore Sigma), 100 ng/mL recombinant human FGF2 (BioPioneer), 50 ng/mL recombinant human EGF (R&D Systems), 50 ng/mL recombinant human HGF (Stem Cell Technologies), and 20 ng/mL Noggin (R&D System). Cells were passaged for 5 times to derive primed-PSC derived TSC (pTSC).

Naïve-iPSCs were derived using our previously published protocol.^97^^,^^98^ In brief, primed iPSC were seeded on a MEF feeder layer in mTeSR Plus under 5% O_2_ and 5% CO_2_. Two days later, media was switched to 5i/L/A, then after 7 to 10 days, cells were passaged using TrypLE Express on a MEF feeder layer under 5% O_2_ and 5% CO_2_. Cells were passaged for 6-8 times to establish naïve iPSC. Subsequently, cells were single cell dissociated using TryPLE Express, and 0.5-1.0x10^6^ cells/well were seeded in one well of a 6-well plate coated with 5μg/mL Collagen IV initially in hTSC media for 5 passages, then transitioned to iCTB media for an additional 3-4 passages to derive nTSC.^43^

Cell purity was ascertained by flow cytometric analysis of surface expression of CTB marker (EGFR) and EVT marker (HLA-G) to ensure that the cells are over 90% EGFR^+^ and less than 20% HLA-G^+^ cells for both nTSC and pTSC.

#### EVT differentiation from primed and naïve TSC

Differentiation of TSCs into EVT were followed by modified Okae protocol.^35^^,^^41^^,^^42^^,^^52^ Prior to differentiation, TSCs were tested for purity to ensure the starting cells are over 90% EGFR^+^, and less then 20% HLA-G^+^ cells. TSC were dissociated using TrypLE Express, and seeded for 2.0x10^5^ cells per well of 6 well plate on 20 μg/ml fibronectin coated plate in 2 mL EVT basal media [DMEM/F12 no HEPES (ThermoFisher) supplemented with 0.1 mM 2-mercaptoethanol (ThermoFisher), 0.3% BSA, 1% ITS-X (ThermoFisher), 7.5 mM A83-01 (Tocris), 2.5 mM Y27632(Selleck Chemicals)] supplemented with 4% KSR (ThermoFisher), 100 ng/mL NRG1 (Abcam), and 2% Matrigel (Corning). On day 3, the media were replaced with EVT basal medium supplemented with 4% KSR, and 0.5% Matrigel. On day 5, cells were collected for flow cytometric analysis, RNA-seq, and WGBS.

### Method details

#### Flow cytometric analysis

Flow cytometry was conducted using live cells. Cells were collected and incubated at room temperature for one hour in FC buffer (0.5% BSA and 1% FBS in PBS) with an APC conjugated mouse anti-human EGFR antibody (clone AY13, BioLegend) and PE-conjugated mouse anti-HLA-G antibody (MEM-G/9, ExBio). APC-conjugated mouse IgG (cloneMOPC-21, BioLegend) and PE-conjugated mouse IgG (cloneMOPC-21, BioLegend) were used as isotype IgG controls. Cells were washed 3 times with FC buffer and analysis was carried out using a BD FACS-Canto Flow Cytometer.

#### Cell proliferation assay

Click-iT EdU Alexa Fluor 488 Flow Cytometry Assay kit (ThermoFisher) was used. TSCs were plated for 5.0x10^4^ cells/well in a 12 well plate coated with collagen IV (Millipore Sigma, 5 μg/mL), one day before the assay. The following day, cells were treated with 10 μM EdU for 2 hours. Cells were then dissociated using TryPLE Express (ThermoFisher), and performed fixation and staining according to the manufacture protocol. and carried out flow cytometric analysis using a BD FACS-Canto Flow Cytometer.

#### Invasion assay

EVT differentiation was performed as detailed in the above. On day 6, cells were replated onto 100 μL of 0.3 mg/mL Matrigel coated Transwell inserts (8.0 μm pore membrane insert in 24-well plate, Corning), for 2.5x10^4^ cells/insert resuspended in 200μL EVT basal media with 0.5% Matrigel in the upper chamber of the insert, then placed in the 24 well plate containing 500 μL of EVT basal medium supplemented with 4% KSR, and 0.5% Matrigel. After 2 days, cells in the upper surface of the membrane were removed with a cotton swab. Cells at the bottom of the membrane were fixed with 4% PFA, and stained with DAPI (ThermoFisher) for the nuclear counting. Cells were imaged at 20x across 15 areas throughout the membrane using a Leica DMI6000B inverted fluorescence microscope. Quantification of invaded cells was performed using Image J^92^ macro with Analyze particle function.

### Immunocytochemistry

Cultured TSCs were fixed in ice-cold 4% paraformaldehyde in PBS for 10 minutes. Cells were then washed using PBS and blocked with a buffer consisting of 10% normal goat serum (Jackson Labs), 5% BSA (Gemini Bio-Products), and 0.25% Triton-X in PBS for one hour. Cells were stained with the following primary antibodies in blocking buffer overnight at 4°C: mouse anti-p40 (delta-N isoform of TP63) antibody (Biocare Medical), and rabbit anti-KRT7 antibody (Abcam). Cells were washed and incubated with Alexa 488- or Alexa 594-conjugated goat anti-mouse and goat anti-rabbit secondary antibodies for one hour at room temperature in the dark. Cells were counterstained with DAPI (Invitrogen), then visualized using a Leica DMI6000B inverted fluorescence microscope.

#### Tumor formation assay

Tumor formation assays was performed under the Institutional Animal Care and Use Committee (IACUC) at UC San Diego (#S09090). Naïve- and primed-iPSC derived TSCs were grown to 90% confluence in iCTB medium (as described above). 1.0 x 10^7^ cells were resuspended in 150μL of a 1:2 mixture of Matrigel and iCTB medium, and subcutaneously injected into the flank or hindleg of 8-12-week-old male NOD-SCID mice (JAX Stock No: 005557). Tumor growths were collected 7-10 days after injection. The tumors were fixed in 10% neutral-buffered formalin overnight at 4°C, then processed and embedded in paraffin. H&E staining was performed on 5-micron sections of these formalin-fixed, paraffin-embedded tissues at the UC San Diego Advanced Tissue Technology Core lab. Slides were analyzed by conventional light microscopy on an Olympus BX43 microscope.

#### RNA isolation, cDNA preparation, quantitative real-time PCR, and RNA sequencing

Total RNA was isolated using mirVana RNA Isolation Kit (Ambion). RNA concentration was measured using Qubit RNA BR assay kit (ThermoFisher). RNA integrity was checked using RNA 6000 Nano chip read by a 2100 bioanalyzer (Agilent). All samples had a RIN above 8.0. cDNA was prepared from total RNA using the Primescipt RT-Kit (Takara bio). Quantitative real-time PCR (qPCR) was performed using TB GREEN (Takara bio) on a QuantStudio 5 thermocycler (ThermoFisher). The primer sequences used are TP63 (ΔNp63 isoform; F 5’-CTG GAA AAC AAT GCC CAG A -3’, R 5’-AGA GAG CAT CGA AGG TGG AG -3’), CDX2 (F 5’-TTC ACT ACA GTC GCT ACA TCA CC -3’, R 5’-TTG ATT TTC CTC TCC TTT GCT C -3’), L19 (F 5’ -AAA ACA AGC GGA TTC TCA TGG A- 3’, R 5’ -TGC GTG CTT CCT TGG TCT TAG- 3’). Relative expression of each transcript was calculated using ΔΔCT method, normalized to L19 rRNA. For miRNA qPCR, cDNA was made using TaqMan Advanced miRNA cDNA Synthesis Kit (cat#: A28007), then qPCR was performed using Applied Biosystems TaqMan Fast Advance Master Mix (cat #4444556) with the following probes (cat#: A25567): hsa-miR-517a-3p (479485_mir), hsa-miR-517-5p (478980_mir), hsa-miR-525-3p (478995_mir), hsa-miR-526b-3p (478996_mir), and housekeeping hsa-miR-103a-3p (478253_mir). Samples were run on a QuantStudio5 384 thermocycler for 55 cycles. Relative expression was calculated using ΔΔCT method, normalized to miR-103a-3p.

RNA-seq libraries were prepared using the TruSeq Stranded Total RNA Sample preparation kit with Ribo-Zero Gold (Illumina) at the IGM Genomics Center, University of California, San Diego, La Jolla, CA. Libraries were pooled and sequenced on NovaSeq 6000 S1Flow Cell (Illumina) to an average depth of 28 million uniquely mapped reads. Quality control was performed using multiQC (v. 1.6).^95^ Reads were mapped to GRCh38.p10 (GENCODE release 26) using STAR (v. 2.7.3a)^96^ and annotated using featureCounts (subread v.1.6.3, GENCODE release 26 primary assembly annotation).^91^ The STAR parameters used were: --runMode alignReads --outSAMmode Full --outSAMattributes Standard --genomeLoad LoadAndKeep --clip3pAdapterSeq AGATCGGAAGAGC --clip3pAdapterMMp 1. The featureCounts parameters were: -s 2 -p -t exon -T 13 -g gene_id. ENSEMBL genes without at least three samples with 10 or more reads were removed from analysis. Normalization and differential expression analysis were performed using the R (v. 4.2.1)^99^ package DESeq2 (v. 1.36.0).^89^ BiomaRt (v. 2.42.1) was used to convert Ensembl gene ID’s to HUGO gene names,^85^ and gene set enrichment analysis was done using the R(v. 4.2.1)^99^ package FGSEA (v. 1.22.0).^100^ Network analysis was done using Cytoscape (v.3.8.0) and the plugin stringApp (v.1.5.1). Following the construction of the String PPI’s, the networks were clustered using MCL clustering with the clusterMaker2 application (v.1.3.1) with the assumption that the edges were undirected and loops were adjusted before clustering. Data visualization was done in R (v. 4.2.1)^99^ using the package ggplot2 (v3.4.2)^101^^,^^102^ and Qlucore Omics Explorer (v3.6) (Qlucore).

#### Whole genome bisulfite sequencing and analyses

DNA was isolated using the DNeasy kit (Qiagen) and quantified using the Qubit dsDNA BR assay kit (ThermoFisher). WGBS library was prepared using Accel-NGS Methyl-seq kit (Swift Biosciences) and sequenced at 15X coverage using paired-end 100 bp reads on a NovaSeq 6000 S4 Flow Cell (Illumina) by the IGM Genomics Center, University of California, San Diego, La Jolla, CA. Genomic DNA was spiked with 0.5% unmethylated lambda phage DNA (Promega) prior to library construction to confirm that bisulfite conversion efficiency was above 99% for each sample. Following sequencing, reads were aligned to GRCh38 using the WGBS pipeline CpG_Me.^86^^,^^88^^,^^95^^,^^103^ Briefly, reads were first adapter trimmed using Trim Galore (v. 0.6.7) and Cutadapt (v. 1.18)^88^ with a maximum error rate of 0.1 and stringency of 1 bp and 10 bp were trimmed off both reads 5’ end and 20 bp off the 3’ end of read one and 10 bp off the 3’ end of read 2. Next the reads were screened for contamination (mouse and lambda phage) using FastQ Screen (v. 0.14.0).^90^ Following the screen, the reads were aligned to the reference genome (hg38) using Bismark (v. 0.23.1)^86^ and Bowtie2 (v. 2.3.4.1)^87^ with the specified options: -q -N 1 –score-min L,0,-0.2 –ignore-quals --no-mixed --no-discordant –dovetail –maxins 500. The reads were then deduplicated using Picard Tools and the methylation level of each CpG site was calculated using the Bismark methylation extractor and only called if it was covered by greater than 5 reads. Annotations of genomic regions were downloaded from ENCODE through CpGtools.^56^ Promoters were defined as regions marked by H3K4Me3 and DNA hypersensitivity or 2,000 bp upstream and 200 bp downstream from transcription start sites. Annotated regions of interest with methylation differences of 20% or more were considered differentially methylated.

### Quantification and statistical analysis

#### Statistical analysis

Bar graph display mean ± standard deviation of triplicates as stated. Box plots show median with the center line, mean as the cross mark, with the box indicating the upper and lower quartile, the whiskers indicating maximum and minimum values, and outlier/single data point being marked as circles. Shapiro-Wilk test was used for test of normality and statistical analysis was done using linear mixed model for parametric data, and quantile regression analysis for non-parametric data to account for technical and biological replicates using R (v. 4.2.1)^99^ package lmerTest (v. 3.1-3)^93^ and lqmm (v. 1.5.8).^94^ ∗ displays statistically significant values (as indicated in the figures). Differential expression analysis was performed using DESeq2 and an adjusted p-value < 0.05 was considered differentially expressed.

## Supplemental information

Document S1. Figures S1–S5 and Tables S1 and S5

Table S2. List of EVT specific genes in Figure 3C that were differentially expressed between the EVT state and the corresponding TSC state (Adjusted p-value < 0.05)

Table S3. List of commong EVT specific genes in Figure 3E (Adjusted p-value <0.05), related to Figure 3D

Table S4. List of genes that are down- and up-regulated in PE pEVT shown in Figure S3C (Adjusted p-value <0.05)
