# Supplementary material for: iPSC-based modeling of preeclampsia identifies epigenetic defects in extravillous trophoblast differentiation
Source: iScience. 2024 Mar 25;27(4):109569. doi: 10.1016/j.isci.2024.109569 (PMC11016801; doi:10.1016/j.isci.2024.109569)
Supplement: Document S1. Figures S1–S5 and Tables S1 and S5 [file mmc1.pdf]

iScience, Volume *27*

Supplemental information

iPSC-based modeling of preeclampsia identifies epigenetic defects in extravillous trophoblast differentiation

Robert Morey, Tony Bui, Virginia Chu Cheung, Chen Dong, Joseph E. Zemke, Daniela Requena, Harneet Arora, Madeline G. Jackson, Donald Pizzo, Thorold W. Theunissen, and Mariko Horii

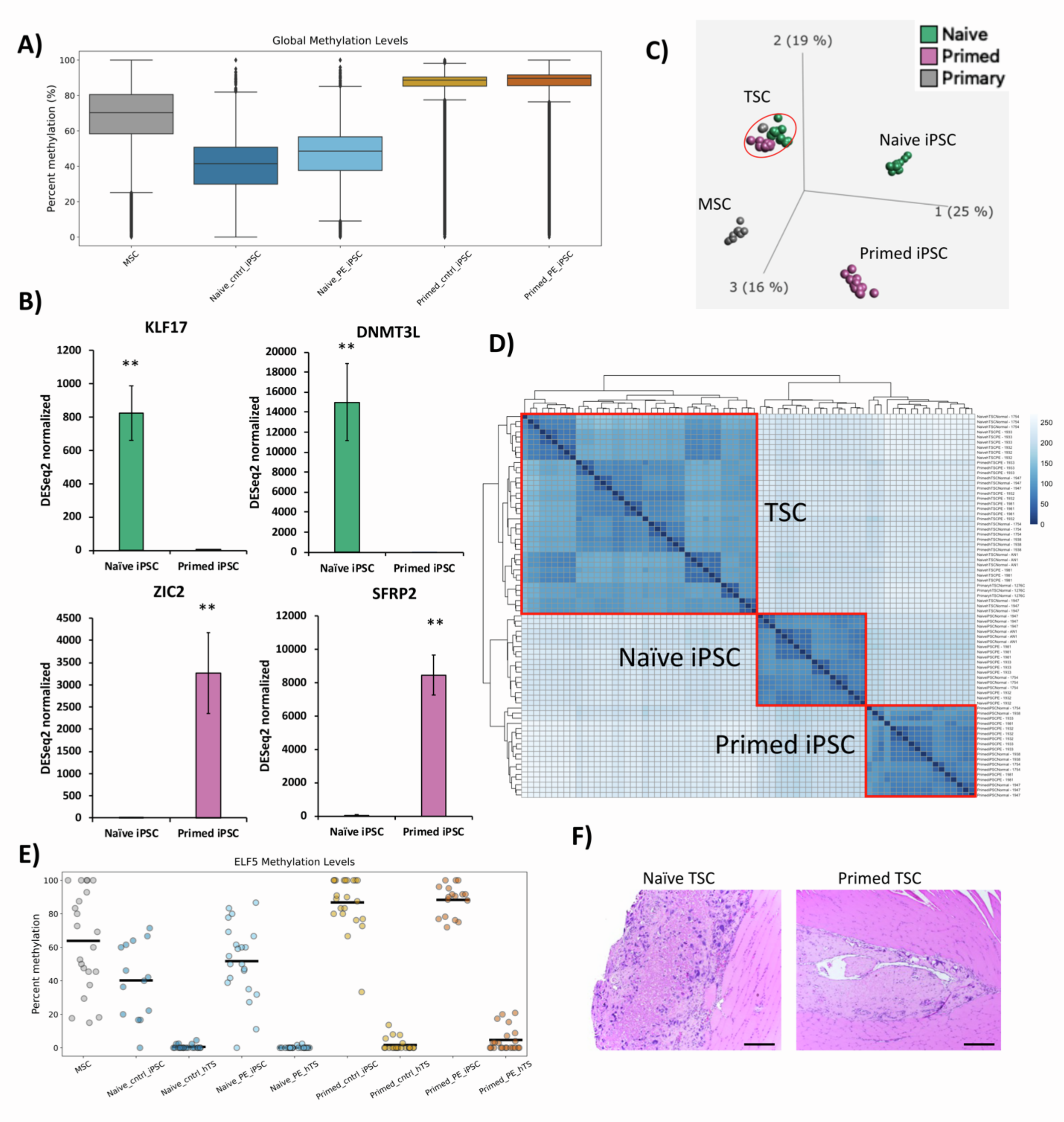

Figure S1 TSC derivation of PE- and control-iPSC from both primed and naïve state pluripotent stem cells, related to Figure 1.

A) Boxplot displaying percent methylation of the global DNA methylation levels of parental MSC, naïve- and primed-iPSC. **B)** Bar graph displaying gene expression levels from normalized values for naïve PSC markers (KLF17 and DNMT3L) and primed PSC markers (ZIC2 and SFRP2). Bar graph display mean ± standard deviation of triplicates. **adjusted p-value <0.01 **C)** PCA plot displaying MSC, primary TSC (1276C)[52], "naïve- and primed-iPSC, and "naïve"- and primed-TSC. Both naïve and primed TSC clustered together indicating the similar gene expression profile in both naïve- and primed-iPSC derived TSC. **D)** Heatmap of Euclidian distance between samples on DESeq2 normalized gene expression, and found that both naïve and primed TSC cluster together with primary TSC.

E) ELF5 promoter DNA methylation level of naïve- and primed-TSCs. iPSC lines are hypermethylated, and once the TSC lines are derived, promotor methylation is hypomethylated. **F)** Representative H&E staining from the tumor formation assay collected from the naïve- and primed-TSC generated 10 days post-injection

into NOD-SCID mice. H&E staining shows the trophoblastic tumor growing in the muscle and forming a tumor with a necrotic center. Scale bars: 200 μm.

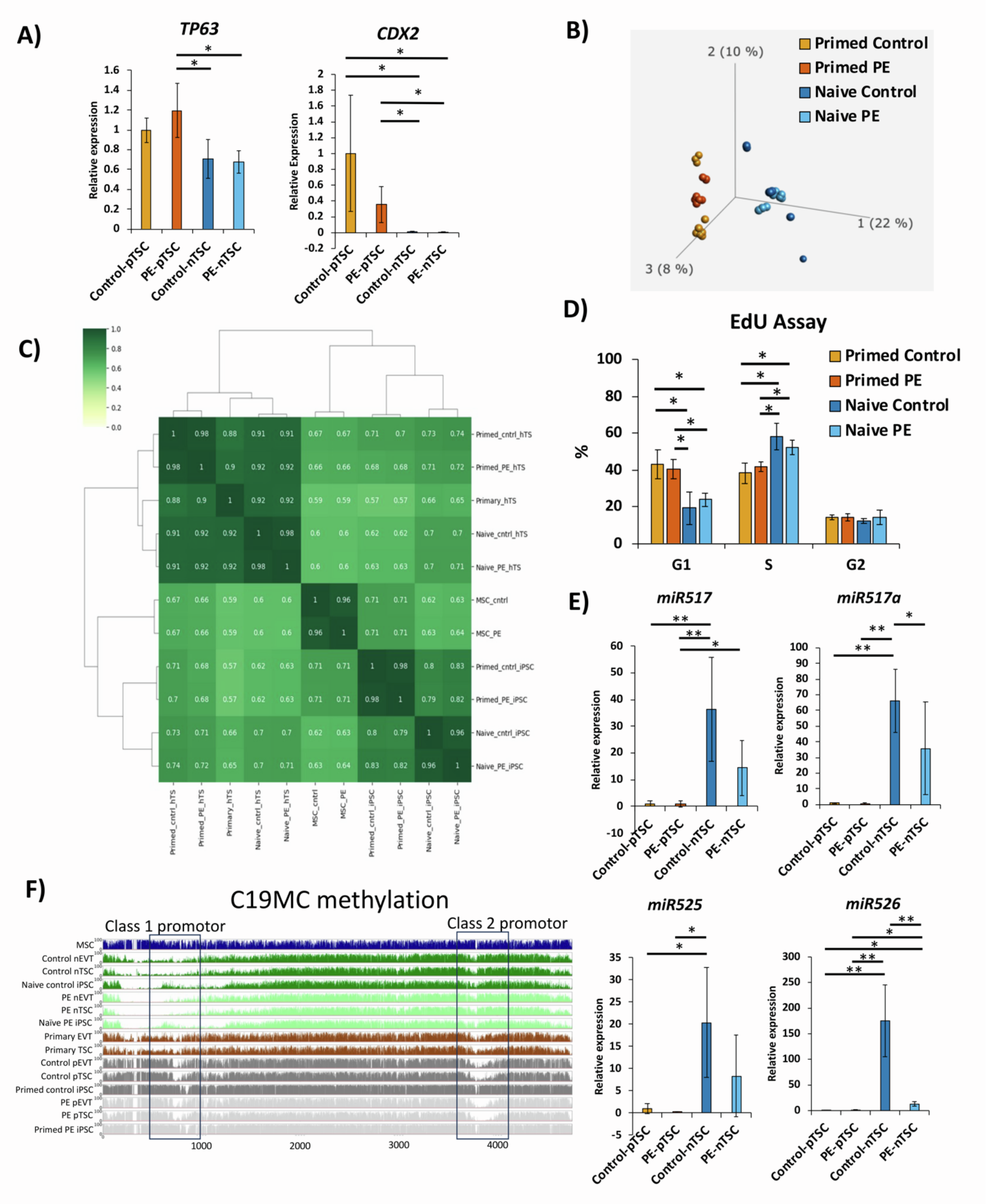

Figure S2. Characterization of TSC from PE and control iPSC, related to Figure 2.
A) Bar graph displaying qPCR of CTB markers *TP63* and *CDX2* of all 4 conditions displayed together. Data were normalized to L19 and shown as fold change over control-pTSC. **B)** PCA plot displaying the 4 conditions of TSC state. Naïve and primed TSC clustered together but not by the disease state indicating the similar gene expression profile within the disease, but the differences appear at TSC derived from different pluripotent state (“naïve” vs. “primed”). **C)** Hierarchical clustered correlation heatmap showing the

Pearson correlation between TSC, iPSC, and MSC lines using methylation sites within the promoter region of 377 CTB-specific genes (Okae et al. 2018). Primary TSC clustered together with naïve- and primed-TSC showing the similarities of promotor methylation level of the CTB specific genes. **D)** Bar graph displaying % cells in each phase of the cell cycle, using the EdU incorporation assay. Naïve-TSC show lower % of cells in G1, and higher % in S phase, compared to primed-TSC. No differences are noted between the disease states. **E)** Bar graph displaying selected gene of C19MC qPCR data, normalized to hsa-miR-103a-3p and shown as fold change over control-pTSC. **F)** DNA methylation patterns in C19MC as indicated. Class 1 and class 2 promotor regions are specified. Bar graph display mean ± standard deviation of triplicates. *$p<0.05$, **<0.01.

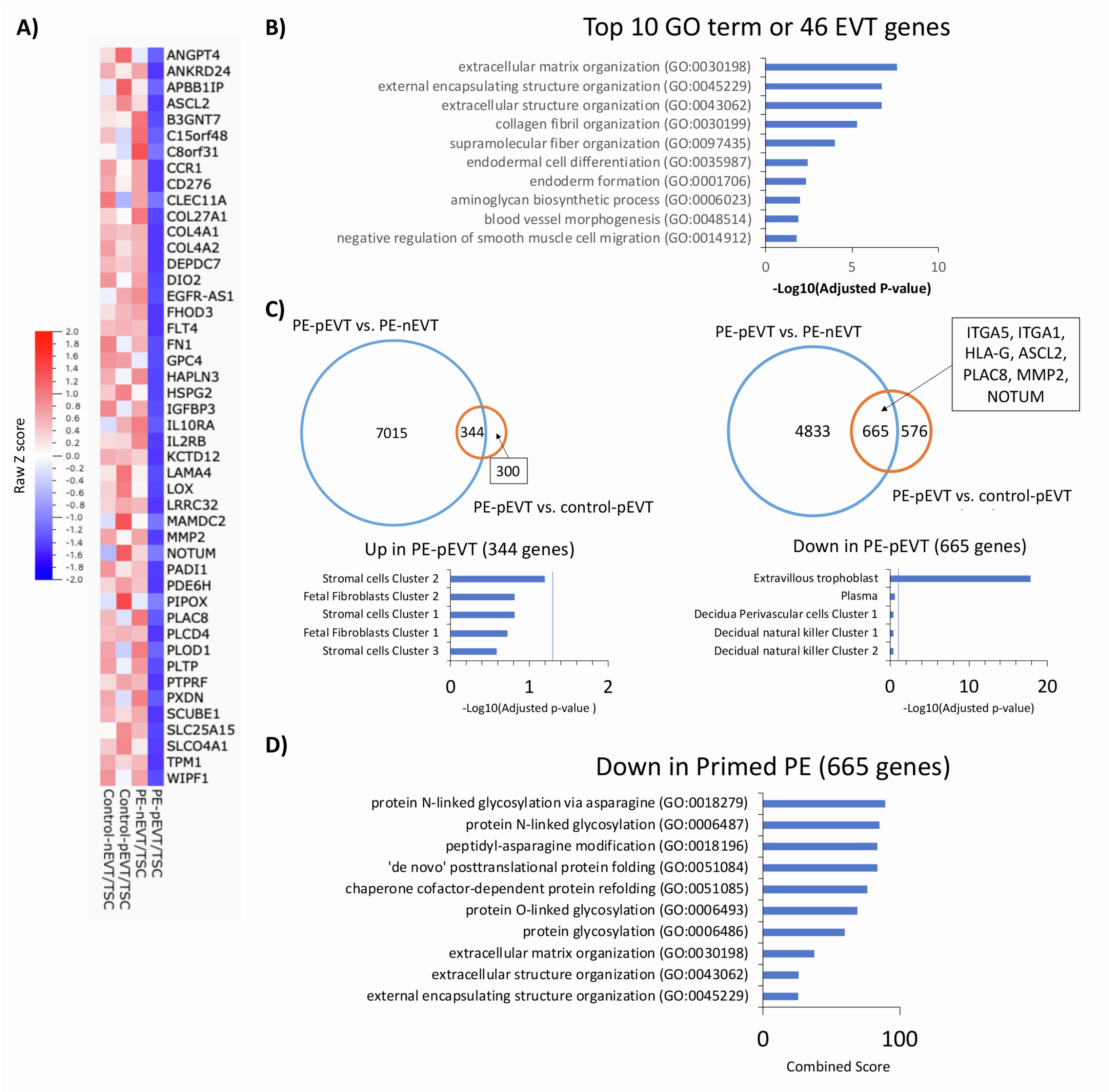

Figure S3. Primed iPSC-derived PE EVT show blunted EVT formation and function, related to Figure 3.

A) Heatmap displaying log2 fold-change of EVT compared to its respective TSC state in the 46 genes that were downregulated in PE-pEVT. **B)** Gene ontology (GO) analysis of 46 genes that lack expression in PE-pEVT, differentiated from PE-pTSC. Bar chart showing top 10 significant GO terms. **C)** Differentially expressed genes specifically up- or down-regulated in PE-pEVT were extracted by taking overlap of the of PE-pEVT vs. PE-nEVT and PE-pEVT vs. PE-nEVT with the same directionality. Genes that are up (n=344) and down (n=665) in PE-EVT were submitted in PlacentaCellEnrich to identify which cell types in placenta are matched to these genes. Bar graph showing the top 5 cell types from the output, with blue vertical line indicating adjusted p-value=0.05, which is at 1.3 in the graph. **D)** Bar graph showing top 10 significant GO terms identified by the Gene ontology analysis of 665 genes which were down regulated in PE-pEVT.

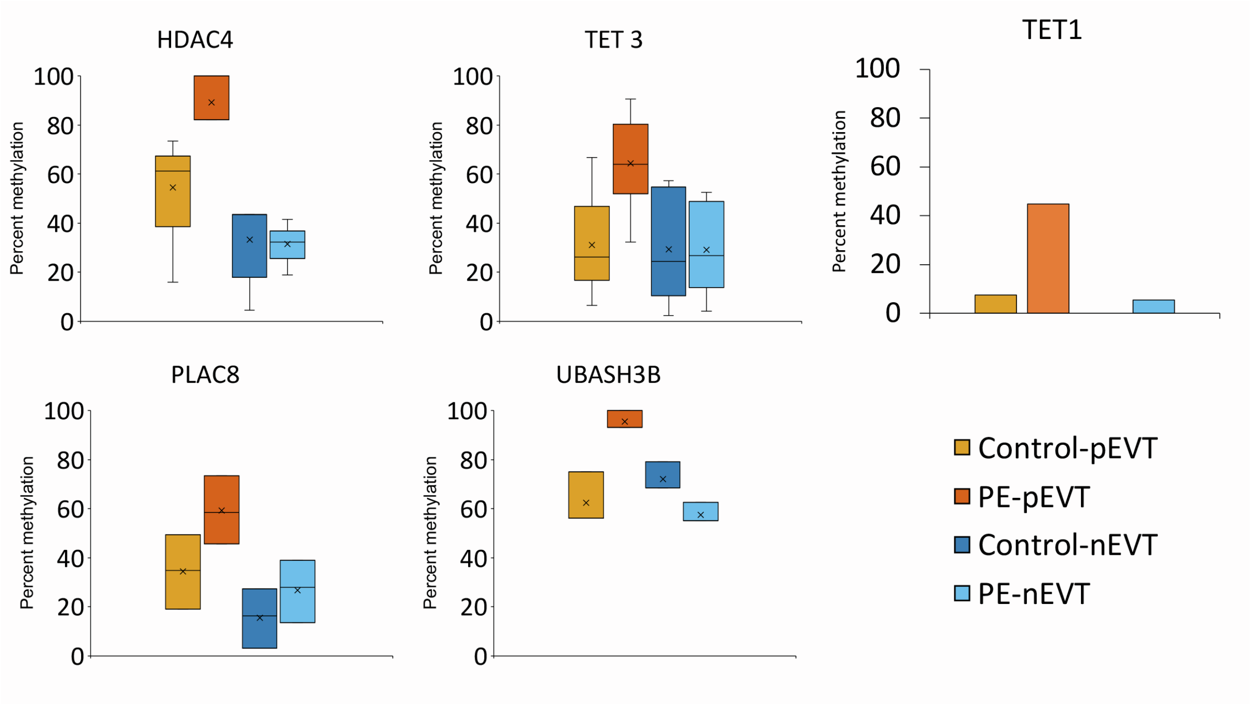

Figure S4. DNA methylation influences phenotypic differences between PE and Control iPSC-derived trophoblast, related to Figure 4B and 4C.
Box plot or bar graph displaying percent DNA methylation of promotor regions (-2,000bp to +200bp from the transcription start site) of genes that are significantly hypermethylated and down-regulated in PE-pEVT. Each graph was plotted by the number of data points available at the promotor region as follows; HDAC4 (n=5), TET3 (n=6), TET1 (n=1), PLAC8 (n=3), UBASH3B (n=3).

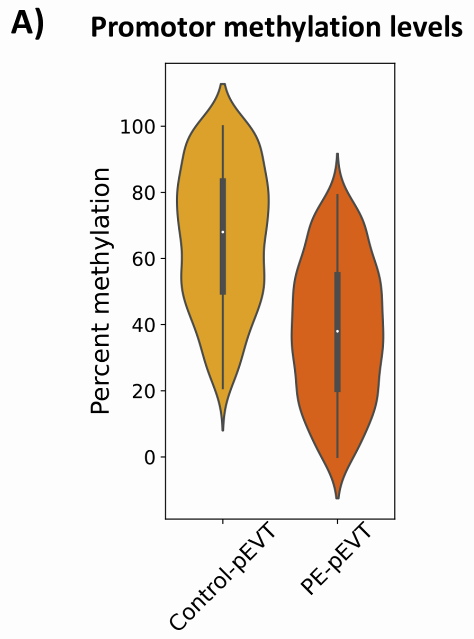

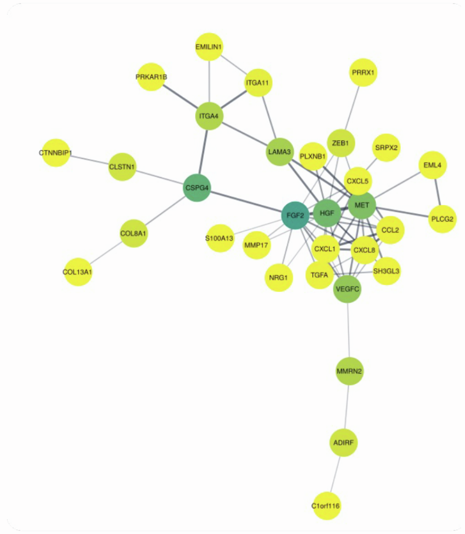

Term	Adjusted p-value
Negative regulation of vascular endothelial growth factor receptor signaling pathway (GO:0030948)	0.045
Regulation of vascular endothelial growth factor receptor signaling pathway (GO:0030947)	0.021

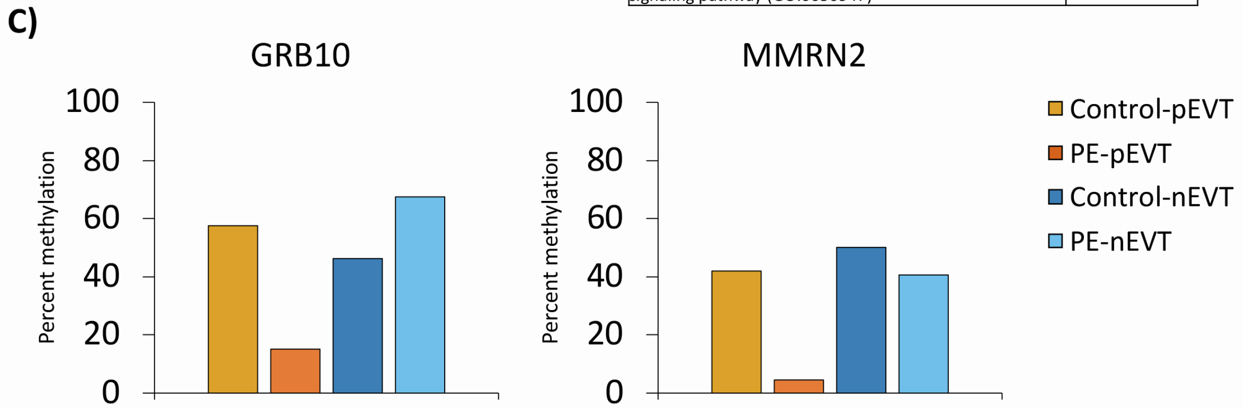

Figure S5. DNA methylation influences phenotypic differences between PE and Control iPSC-derived trophoblast, related to Figure S3C.

A) Violin plot displaying hypomethylation at promotor regions (-2,000bp to +200bp from the transcription start site) of 137 genes that are specifically down-regulated in PE-pEVT, compared to three other conditions. **B)** PPI network analysis showing hub genes specific to 137 genes. Pathways identified form the PPI network analysis are shown in the table. **C)** Bar graph displaying percent DNA methylation of promotor regions (-2,000bp to +200bp from the transcription start site) of genes that are significantly hypomethylated and up-regulated in PE-pEVT. Each graph was plotted by the number of data points available at the promotor region as follows; GRB10 (n=1), MMRN2 (n=1)

Table S1. Number of differentially expressed genes up-regulated in TSC state and % similarities, related to Figure 2.

iPSC vs. TSC	# of CTB specific genes (377 genes from Okae et al. 2018)	% Similarity (n=142)
Primed control	189	75.1
Primed PE	193	73.6
Naive control	201	70.6
Naïve PE	202	70.3

Differentially expressed genes up-regulated in TSC state compared to its respective iPSC state were contrasted to CTB specific genes (Okae *et al*. 2018; n=377 genes). Among these genes, 142 genes were common across the four groups, identifying over 70% similarities among the four groups.

Table S5. List of genes that are up-regulated in gene expression and hypomethylated (at least 20% methylation difference) in PE pEVT. (Related to Figure S5A)

**18 genes that remained after expanding the comparison to all group**
CDC42EP3
PLXNB1
APBB2
PRKAR1B
GRB10
ATXN7L1
DENND3
CYHR1
CAMSAP1
PARD3
MMRN2
IFITM2
LINC01588
CSPG4
SLC9A3R2
SNX29
ZNF385C
CUEDC1
